# The development of novel bioactive porous titanium as a bone reconstruction material

**DOI:** 10.1039/d0ra03202f

**Published:** 2020-06-12

**Authors:** Kazuya Doi, Reiko Kobatake, Yusuke Makihara, Yoshifumi Oki, Hanako Umehara, Takayasu Kubo, Kazuhiro Tsuga

**Affiliations:** Department of Advanced Prosthodontics, Hiroshima University Graduate School of Biomedical and Health Sciences 1-2-3, Kasumi, Minami-ku Hiroshima 734-8553 Japan kazuya17@hiroshima-u.ac.jp +81 82 257 5679 +81 82 257 5677

## Abstract

Porous titanium fabricated by the resin-impregnated titanium substitute technique has good mechanical strength and osteoconduction. The alkali treatment of the titanium surface creates a bioactive surface. Alkali-treated porous titanium is expected to accelerate bone formation. The purpose of this study was to evaluate the bone reconstruction ability of alkali-treated porous titanium. Porous titanium (85% porosity) was treated with an alkali solution (5 N NaOH, 24 h). To assess material properties, we analyzed the surface structure by scanning electron microscopy (SEM) and mechanical strength testing. To assess bioactivity, each sample was soaked in a simulated body fluid (Hank's solution) for 7 days. Surface observations, weight change ratio measurement (after/before being soaked in Hank's solution) and surface elemental analysis were performed. We also designed an *in vivo* study with rabbit femurs. After 2 and 3 weeks of implantation, histological observations and histomorphometric bone formation ratio analysis were performed. All data were statistically analyzed using a Student's *t*-test (*P* < 0.05) (this study was approved by the Hiroshima University animal experiment ethics committee: A11-5-5). Non-treated porous titanium (control) appeared to have a smooth surface and the alkali-treated porous titanium (ATPT) had a nano-sized needle-like rough surface. ATPT had similar mechanical strength to that of the control. After soaking into the Hank's solution, we observed apatite-like crystals in the SEM image, weight gain, and high Ca and P contents in ATPT. There was significant bone formation at an early stage in ATPT compared with that in control. It was suggested that the alkali-treated porous titanium had a bioactive surface and induced bone reconstruction effectively. This novel bioactive porous titanium can be expected to be a good bone reconstruction material.

## Abstract 2

Porous titanium fabricated by the resin-impregnated titanium substitute technique has good mechanical strength and osteoconduction.

## Graphical abstract

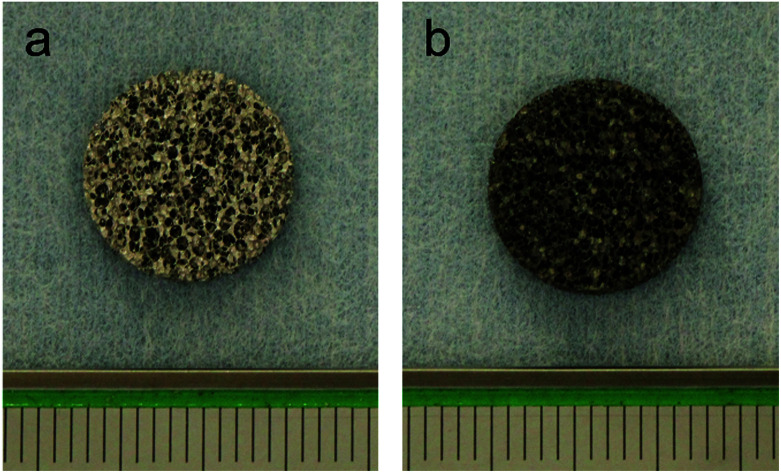

## 1 Introduction

Porous metallic structures are excellent osteoconductive materials and can be used for bone reconstruction owing to their interconnected porous structure, which can promote angiogenesis and migration of bone-producing cells. Among the available porous biomaterials, porous hydroxyapatite is often applied for bone reconstruction. However, it is difficult to apply this material for large bone defects under overload because of insufficient mechanical strength. To overcome these limitations, we chose porous titanium.^1–5^ Porous titanium is widely applied in orthopedic and dental fields because it has good biocompatibility^6^ and can avoid the stress-shielding effects compared to bulk titanium.^7^ The optimal structure for bone formation requires uniform pores in the range of 200–500 μm;^3,5,8^ this enables the colonization of osteoblasts and fibroblasts and vascular ingrowth of osteoconduction. Several methods have been developed for the fabrication of porous titanium structures. We have previously reported a novel porous titanium fabrication method called the resin-impregnated titanium substitution technique; using this technique, we fabricated porous titanium with a similar structure to that of urethane form as base material.^9^ We have successfully used this method to fabricate 65–85% porous titanium with good mechanical strength and osteoconductivity. Using this process, we can fabricate the same structure of titanium as that in a base urethane form. However, porous titanium is not bioactive enough to promote bone formation owing to the classified bioinert material used.^10,11^ Various treatment methods, including grit blasting, acid etching, and titanium plasma spraying, have improved the bioactive topography of titanium.^12–14^ A majority of these techniques are used for dental implants or fixing plates. Physical treatments, such as grit blasting and plasma spraying, limit the effects on the surface of porous titanium. Chemical treatments can be applied to the inside of the porous structure; however, strong acids may corrode the structure, thereby decreasing the mechanical strength of porous titanium.^15,16^ On the other hand, alkali treatment created a rough surface by the formation of sodium hydrogen titanate layers on the titanium surface, which in turn led to a superhydrophilic nanoporous structure on the surface.^16–19^ This surface structure is good for bone formation and inflammation-immunological balance.^20^ Moreover, alkali treatment does not affect the mechanical strength and structure.^16^

We hypothesized that treating porous titanium with an alkali can help generate a bioactive surface, maintain the structure, and promote bone formation. Therefore, in this study, we aimed to evaluate bone reconstruction using alkali-treated porous titanium (ATPT).

## 2 Experimental

### 2.1. Sample preparation

**Fig. 1:**
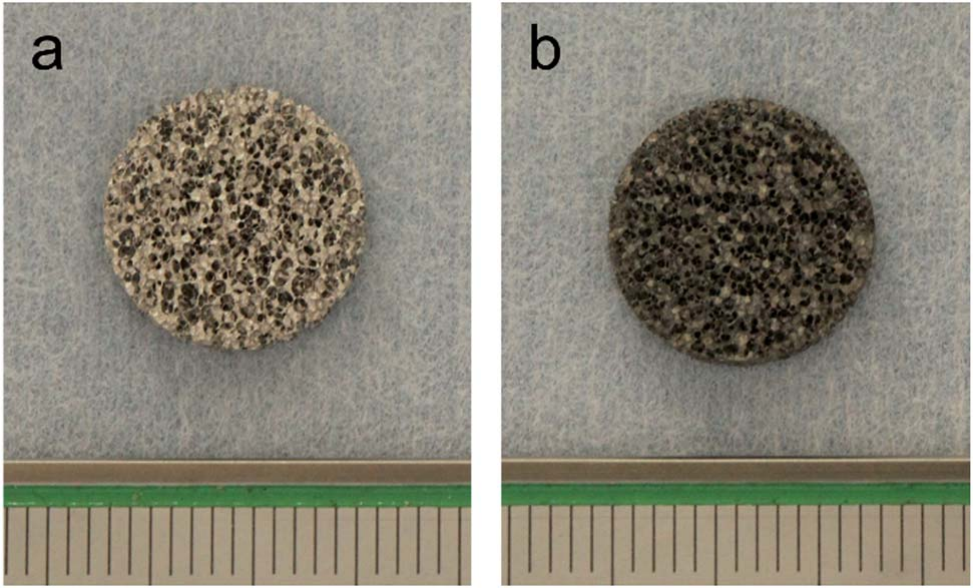
Samples. (a) Control, (b) ATPT.

Porous pure titanium discs (*φ* 13 mm × *H* 2 mm, 85% porosity) were used in this study. Samples of porous titanium, with 85% interconnected porous structures, were fabricated using a resin-impregnated titanium substitution method, as described previously.^9^ The surface treatment process was as per our previous study.^16,19^ For non-treated porous titanium (control), samples were washed with an ultrasonic cleaner using acetone and distilled water for 1 h each and dried overnight in a 37 °C oven (Fig. 1a). For ATPT, discs were washed with acetone and distilled water followed by immersion in 20 mL per disc of 5 N NaOH and incubated at 60 °C for 24 h with gentle shaking. After incubation, the discs were washed with distilled water for 1 h and dried in a 37 °C oven (Fig. 1b).

### 2.2. Assessment of material properties

#### 2.2-1. Observation of the surface structure

The surface structure of each disc was determined by scanning electron microscopy (SEM, JSM-7200F, JEOL Ltd. Tokyo, Japan). Each sample was sputter-coated with platinum, attached to a sample stage using carbon adhesive tape, and imaged.

#### 2.2-2. Measurement of mechanical strength

Each sample was set on the stage and the crosshead speed was 0.5 mm min^−1^. The first peak load value (*N*) of each sample was determined as compression strength (AUTO GRAPH AGS-X, SHIMADZU, Kyoto, Japan). The compression strength was then recorded as the mechanical strength (MPa) (*n* = 4).

### 2.3. Evaluation of bioactivity

Hank's balanced salt solution without phenol red (Hank's solution) (HBSS10-527F, Lonza, Walkersville, USA) was used for the evaluation of bioactivity. Each sample was placed in an untreated plastic 24-well plate and incubated with 2.5 mL of Hank's solution at 37 °C for 7 days. The solution was replaced every day. After immersion in Hank's solution, the samples were removed from the solution, gently washed with distilled water, and dried in a 37 °C oven.

#### 2.3-1. Observation of the surface composition

After sputtering with platinum, the Hank's solution-soaked samples were attached to a sample stage using a carbonate adhesive tape and imaged by SEM.

#### 2.3-2. Weight change ratio before and after Hank's solution treatment

The weight of the samples was measured using an electronic balance (AUW120D, SHIMADZU, Kyoto, Japan) before and after soaking in Hank's solution. The weight change ratio was calculated by after immersion weight/before immersion weight (×100%) (*n* = 4).

#### 2.3-3. Surface element analysis

Samples with or without soaking were fixed on a stage. Subsequently, we performed local electron probe microanalysis (EPMA-1720H, SHIMADZU, Kyoto, Japan). The conditions used included an acceleration voltage of 15 kV, a beam current of 100 nA, and a beam size of 100 μm.

### 2.4. *In vivo* studies

**Fig. 2:**
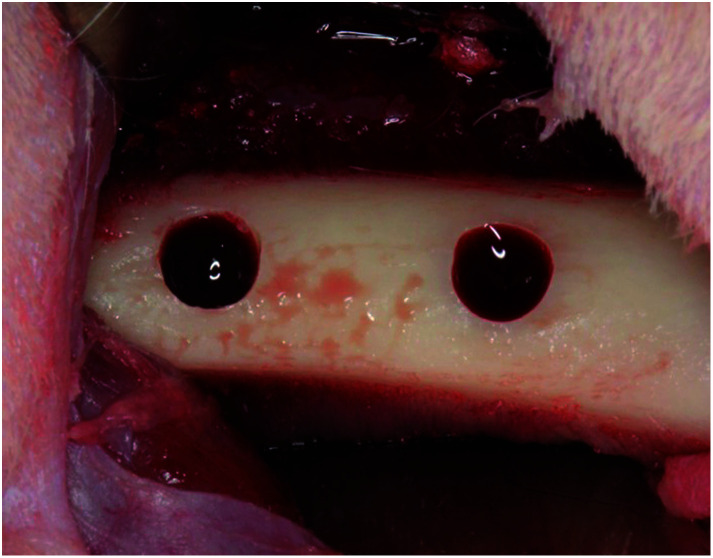
Implantation. Two bone defects (diameter 3 mm, length 3 mm) on the rabbit femurs on both sides were prepared. Short type samples of each sample were randomly placed in the bone defects.

This study was approved by the Research Facilities Committee for Laboratory Animal Science, Hiroshima University School of Medicine (approval no. A11-5-5). Three New Zealand white rabbits (male, 17 week-old, 3.0–3.5 kg) were used. The surgical procedures were performed on rabbits under general anaesthesia with sodium pentobarbital (10 mg kg^−1^) and local infiltration anesthesia with 2% lidocaine and 1 : 80 000 noradrenaline. Muscle and periosteal flaps were made on the right femurs and two bone defects (diameter: 3 mm; length: 3 mm) were made. Cylindrical porous titanium was randomly placed in the bone defects (Fig. 2). After 1 week, the same protocol was performed on the left femurs. Two weeks after the second operation, the rabbits were anaesthetized and perfused with 10% neutral formalin *via* the aorta. Femurs were harvested and fixed in 10% neutral formalin for 1 week. Tissue blocks from each bone socket were cut and dehydrated using a series of graded ethanol concentrations and embedded in resins (Technovit 7200VLC, Kulzer, Wehrheim, Germany). The resin blocks were cut in halves and ground to ∼80 μm thickness. The specimens were stained with toluidine blue and images of bone regeneration were digitized and histomorphometrically analyzed using the ImageJ software (National Institutes of Health, Bethesda, MD, USA). The bone formation ratio was calculated as the bone tissue in the pores divided by the total area of the tissue in the pores.

## 3 Statistical analysis

Data were analyzed by one-way analysis of variance followed by the Students' *t*-test (*P* < 0.05) and were expressed as mean ± standard deviation.

## 4 Results

### 4.1. The surface of the titanium discs

**Fig. 3:**
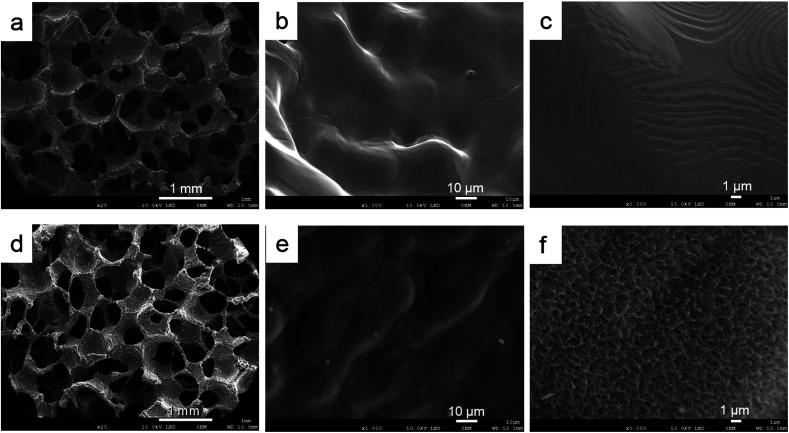
Scanning electron microscopy images of surface porous structures: (a–c) control, (d–f) ATPT. Both samples had similar interconnected porous structures (a and d). Control had a smooth surface (b and c), and ATPT had needle-like and tiny pore structure (e and f).

Based on the low-magnification SEM images, the samples had similar outer structures (Fig. 3a and d). The titanium powder structure could be observed in both groups (Fig. 3b and e). Based on the high–magnification image, the surface topography of the control sample indicated a smooth structure (Fig. 3c). The ATPT sample had nanoscale, needle-like, and tiny pores distributed evenly across its surface (Fig. 3f).

### 4.2. Measurement of mechanical strength

**Fig. 4:**
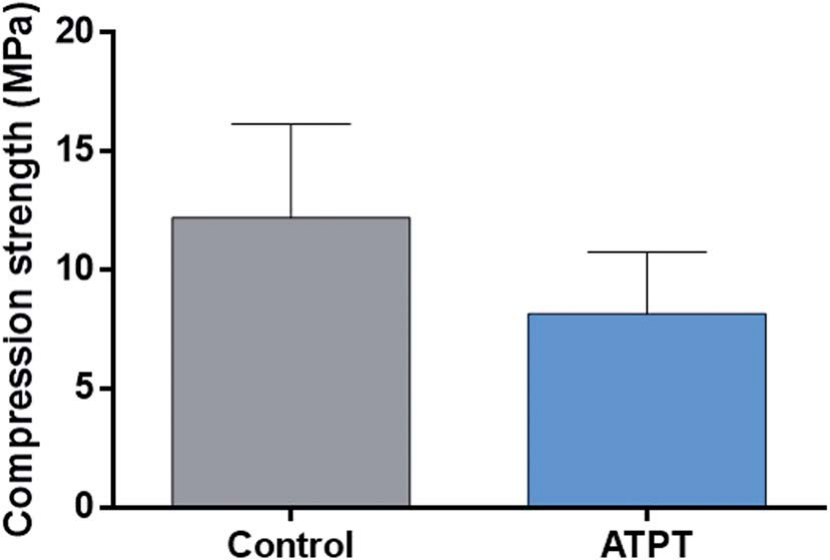
Mechanical strength. There was no significant difference in control and ATPT.

There were no significant differences between the control and ATPT samples (Fig. 4).

### 4.3. Surface observation after soaking in Hank's solution

**Fig. 5:**
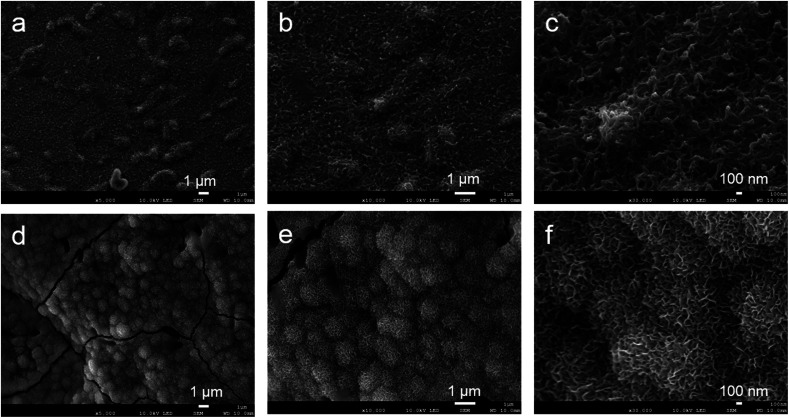
Surface observation after soaking in Hank's solution. (a–c) Control, (d–f) ATPT. Control had several small oval-shaped structures. ATPT had a lot of mature apatite-like structures.

Both the porous titanium samples showed some precipitation on their surfaces (Fig. 5a and d). In the control sample, we observed multiple small oval-shaped structures (Fig. 5b). At high magnification, the image showed a relatively sparse network (Fig. 5c). However, in the alkali-treated sample, there was an abundance of sphere-shaped mature apatite-like structures (Fig. 5e and f).

### 4.4 Weight change ratio before and after Hank's solution treatment

After soaking in Hank's solution, an increase in weight was observed in both groups. ATPT had a higher value compared to that of the control sample (Table 1).

**Table 1:** Weight change ratio before and after Hank's solution immersion

	Change in weight (%)
Control	+0.8 ± 0.2
ATPT	+1.6 ± 0.1^a^

a *P* < 0.05.

### 4.5 Surface elements

Before soaking in Hank's solution, 3% C was detected in the control sample, whereas we found peaks for 0.82% C, 27% O, and 1.18% Na for the alkali-treated sample.

After soaking in Hank's solution, the O, Na, P, and Ca contents increased in both the discs. These elements were found in Hank's solution. The P and Ca contents were significantly higher in the alkali-treated sample as compared to those in the control (Table 2).

**Table 2:** Surface elemental analysis

Element	Before SBF		After SBF	
	Control	ATPT	Control	ATPT
C	3.00	0.82	1.90	1.58
O	—	27.00	28.83	33.08
Na	—	1.18	0.88	1.26
P	—	—	2.94	4.77
Ca	—	0.09	2.92	6.26
Ti	91.41	64.64	60.80	44.90

### 4.6. *In vivo* study

**Fig. 6:**
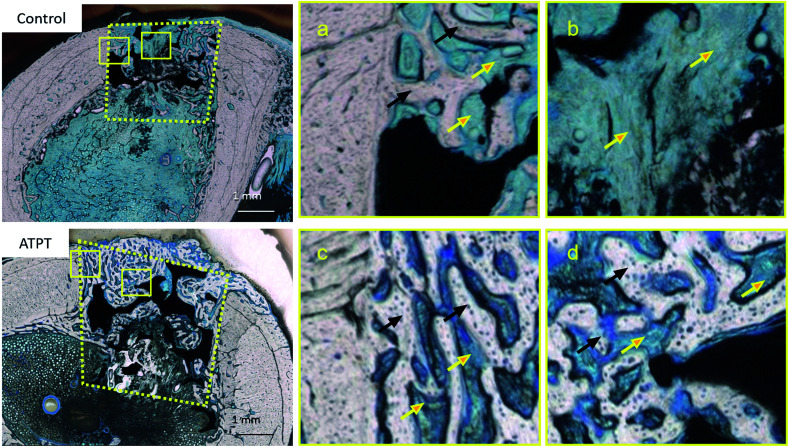
Histological observations with toluidine blue staining at 2 weeks. Control: (a) marginal portion (b) central portion. ATPT: (c) marginal portion (d) central portion. Black arrows indicate newly formed bones. Yellow arrows indicate marrow and connective tissues. In 2 weeks, at the central portion, control was mainly occupied by soft tissues and ATPT had good bone formation.

**Fig. 7:**
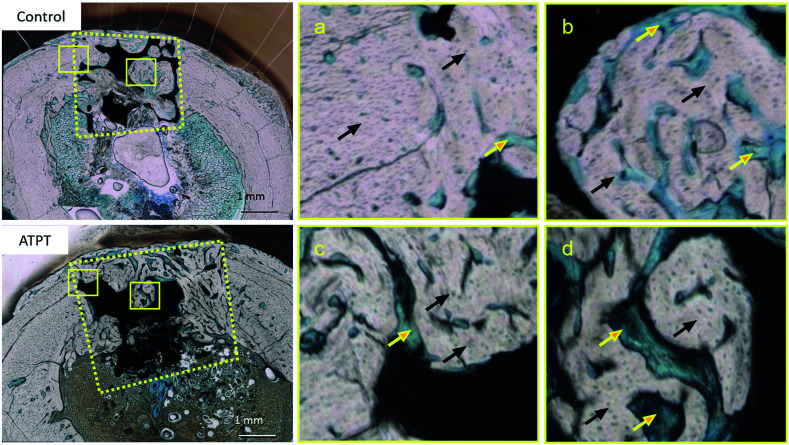
Histological observations with toluidine blue staining at 3 weeks. Control: (a) marginal portion (b) central portion. ATPT: (c) marginal portion (d) central portion. Black arrows indicate newly formed bones. Yellow arrows indicate marrow and connective tissues. In 3 weeks, at the central portion, both samples had good bone formation.

**Fig. 8:**
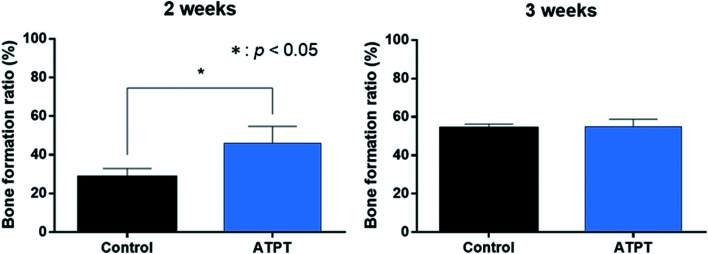
Bone formation ratio. In 2 weeks, ATPT had significantly higher than that of control. In 3 weeks, there were no significant differences.

With respect to histology, there were no signs of inflammation. The black arrows indicate newly formed bones and yellow arrows indicate soft tissues. After 2 weeks, the marginal portions showed continuous bone formation from the parent bone in both groups (Fig. 6a and c). However, in the centre, the control allowed little bone formation that was mainly occupied by soft tissues (Fig. 6b); on the other hand, ATPT showed bone formation in the centre, but the trabecula was thin (Fig. 6d). After 3 weeks, we observed good bone formation from the parent bone in the marginal and central portions of both groups (Fig. 7). The trabecula was thicker than that observed at 2 weeks. Porous titanium and bone were directly connected without the presence of soft tissues. After 2 weeks, the bone formation ratio was higher in the alkali-treated discs as compared to that in the control; there were no significant differences at 3 weeks (Fig. 8).

## 5 Discussion

ATPT was bioactive and allowed a good amount of bone formation. In this study, we used an NaOH solution to modify the surface of porous titanium.

To accelerate bone formation, numerous methods have been reported such as using growth factors and cells. There are also various methods to obtain modified surfaces, such as UV irradiation, blast-etching, and chemical treatments.^12–14,16^ Surface roughness, topography, and chemistry are crucial factors for bone formation.^21^ However, growth factors and cells have a risk of immune response and allergies. UV light and blast powders do not reach the inner pores since the porous metal is three dimensional. Therefore, we used chemical surface treatment, which is more simple, safe and effective, for porous titanium.

Titanium is a bioinert material that forms a film of oxide on the surface (for passivation) and is resistant to corrosion.^22^ Strong acidic solutions of up to pH 3 can corrode titanium.^17^ Thus, acid treatment reduces the mechanical strength of porous metals. In contrast, alkali treatment can modify the surface of titanium without corroding it as it does not alter its outer structure and mechanical strength.^16^ Thus, we selected alkali treatment using an NaOH solution to modify the titanium surface.

SEM images showed a needle-like porous structure of the sodium titanate layer on the surface of ATPT. We performed a Hank's solution immersion test to evaluate this bioactivity. The sodium titanate layer is bioactive in the human body owing to the crystallized apatite formed on the titanium surface.^23^

Hank's solution is used for the apatite precipitation observation test as well as SBF.^24,25^ This sodium titanate layer is negatively charged in Hank's solution; thus, it binds positively charged calcium ions and negatively charged phosphate ions. The mechanism of the Ca–P compound formation is as follows: Na^+^ ions on the titanium surface are exchanged with H_3_O^+^ ions from the Hank's solution, and the Na^+^ ions are released. Then, Ti–OH groups are formed on the surface, which are involved in the nucleation of the Ca–P compounds. This explains the increase in the Ca and P contents and the Ca/P ratio of ATPT after immersion in Hank's solution as compared to that in the control. After being immersed in Hank's solution, the obtained SEM images showed mature apatite-like spheres in ATPT as compared to that in the control. Also, the weight increase ratio of ATPT after immersion in Hank's solution was significantly higher than that in the control. This result is explained by the difference in the formation ability of the calcium phosphate composite deposited on the surface in Hank's solution. These results indicate that the crystallization of apatite is stimulated on the surface of ATPT. The deposition of calcium phosphate indicated bioactivity. There are reports that calcium phosphate crystals affect hard tissue induction.^26,27^

Our results showed that there were no inflammation signs and allergic signs in animals; therefore, each porous titanium sample indicated the immunological biocompatibility of materials.^28^ ATPT accelerated apatite formation, indicating high bioactivity in the body. Both groups of discs after 2 weeks of implantation demonstrated newly formed bones from the existing bones in the marginal portion. It is known that pore size affects osteoconduction. Our titanium samples had a pore size of 200–300 μm. There are reports that pore sizes of 200–500 μm are optimal for the colonization of osteoblasts and fibroblasts or vascular ingrowth of new bones. We speculated that similar bone formation in the marginal portion was due to osteoconduction from the surrounding bone tissues.^8,9^ In 2 weeks, both groups indicated bone formation at the marginal portion. However, only ATPT enabled bone formation in the central portion. Bone formation was observed in the central portion of the ATPT sample, whereas minimal tissues were detected using the control. The central portion of the bone defects is generally far from the surrounding bones and the early stages of bone formation are affected by osteoinduction rather than osteoconduction. This agrees with the results of apatite formation in Hank's solution and surface element analysis. Since the alkali-treated titanium surface was rough and bioactive, bone formation was stimulated by activating the osteoblasts and apatite formation.^6^ Therefore, the bone was formed in the central portion 2 weeks after implantation and the bone formation ratio was higher using ATPT as compared to that for the control.

In 3 weeks, we observed good bone formation in the central portion of both groups and this was in accordance with our histomorphometric analysis. We considered that the bone was formed by the osteoconduction of the surrounding bones at 3 weeks because of the socket-shaped bone defect.

## 6 Conclusion

We demonstrated that ATPT is more bioactive and forms apatite compared to untreated titanium. Thus, this newly developed bioactive porous titanium accelerates bone formation and can be used as a bone reconstruction material.

## Conflicts of interest

There are no conflicts to declare.

## Supplementary Material
